# Exploring the pedagogical design features of the flipped classroom in undergraduate nursing education: a systematic review

**DOI:** 10.1186/s12912-021-00555-w

**Published:** 2021-03-22

**Authors:** Punithalingam Youhasan, Yan Chen, Mataroria Lyndon, Marcus A. Henning

**Affiliations:** 1 Centre for Medical and Health Sciences Education, Faculty of Medical and Health Sciences, The University of Auckland, Auckland, New Zealand; 2 Department of Medical Education & Research, Faculty of Health-Care Sciences, Eastern University, Sri Lanka, Batticaloa, Sri Lanka

**Keywords:** Flipped classroom, Blended Learning, Inverted classroom, Nursing education, Systematic review, Design principles

## Abstract

**Background:**

In recent years, technological advancement has enabled the use of blended learning approaches, including flipped classrooms. Flipped classrooms promote higher-order knowledge application – a key component of nursing education. This systematic review aims to evaluate the empirical evidence and refereed literature pertaining to the development, application and effectiveness of flipped classrooms in reference to undergraduate nursing education.

**Methods:**

A PRISMA systematic review protocol was implemented to investigate the literature pertaining to the development, implementation and effectiveness of flipped classroom pedagogy in undergraduate nursing education. Seven databases (Scopus, PsycINFO, CINAHL, ERIC, MEDLINE, Cochrane, Web of Science) were utilised to survey the salient literature. Articles were appraised with respect to their level of evidence, the origin of study, study design, the aims/s of the study, and the key outcomes of the study. A qualitative synthesis was then conducted to summarise the study findings.

**Results:**

The initial search identified 1263 potentially relevant articles. After comprehensively reviewing the initial catchment using several analytical phases, 27 articles were considered for the final review, most of which were conducted in the USA and South Korea. A range of research designs were applied to measure or discuss the outcomes and design features of the flipped classroom pedagogy when applied to undergraduate nursing education. The review indicated that a common operational flipped classroom model involves three key components, namely pre-classroom activities, in-classroom activities and post-classroom activities, guided by two instructional system design principles. The review predominantly identified positive learning outcomes among undergraduate nursing students, after experiencing the flipped classroom, in terms of skills, knowledge and attitudes. However, a few studies reported contrasting findings, possibly due to the incompatibility of the flipped classroom pedagogy with the traditional learning culture.

**Conclusions:**

Current evidence in this systematic review suggests that incorporating the flipped classroom pedagogy could yield positive educational outcomes in undergraduate nursing education. There are promising pedagogical models available for adapting or developing the flipped classroom pedagogy in undergraduate nursing education.

## Background

Globally, nursing educational institutions are taking steps forward in redesigning their curricula to align them with modern pedagogy to enhance student-centred learning [1]. This curricular reform is concerned with fulfilling the educational needs of the new technological era, which generates exposure to a variety of information, advanced communication technology, and diverse learning methods [2]. As a result, blended learning has become part of this curricular reform. Blended learning is a broad pedagogical approach, which encompasses a combination of face-to-face and online teaching to promote student-centred learning [3]. One of the blended learning innovations is the notion of the flipped classroom [4], referred to as “a hybrid approach to learning, using technology to move the classroom lecture to homework status and using face-to-face classroom time for interactive learning” [5]. The rule of thumb of the flipped classroom is redesigning the face-to-face classroom as an interactive learning environment where higher-ordered learning takes place, while providing traditional pedagogical experiences (of transferring basic information) through pre-class learning activities [4, 6–8]. As such, pre-class learning materials can be provided to closely represent learning in the traditional face-to-face classroom but being delivered electronically or via online media [9]. Therefore, pre-learning materials should be accompanied with teachers’ explanation rather than relying on the sole use of pre-class reading materials [6].

Contemporary nursing care is advancing dramatically due to the need for nursing students to manage problems associated with multiple and complex clinical comorbidities [10]. It was reported in the literature that nursing students experience difficulties in applying learnt knowledge in clinical practice [11]. This necessitated the need for nursing curricular implementers to adopt pedagogies like the flipped classroom to ensure that theoretical concepts were explicitly linked to patient care. As such, the flipped classroom is considered as a new educational paradigm for implementing health professions’ education curricula [6, 12]. While there are increasing applications of the flipped classroom, there is a dearth of evidence evaluating its impact on student learning and curriculum design in undergraduate nursing education [13–15]. The empirical evidence to date has predominantly examined the effectiveness of the flipped classroom through students’ satisfaction and academic performance [13]; however, there is limited evidence to explain the pedagogical design principles of the flipped classroom, which are indispensable to achieving meaningful educational effectiveness [16]. Thus, the following systematic review aims to describe and evaluate research conducted in the area of designing, developing and implementing the flipped classroom, and appraise the educational impact of the flipped classroom approach when applied to undergraduate nursing education.

## Methods

This systematic review was performed in accordance with the PRISMA (Preferred Reporting Items for Systematic Reviews and Meta-Analyses) protocols (Additional File) [17]. PRISMA is an evidence-based system used to guide reporting in systematic reviews and meta-analyses [18]. The protocol was registered with the PROSPERO (International prospective register of systematic reviews) (CRD42020194474, 16th October 2020).

### Systematic literature search

A literature search was conducted utilising seven databases (PsycINFO, CINAHL, ERIC, MEDLINE, Web of Science, Cochrane Library and Scopus) in November 2019. The key concept of the literature search was the term flipped classroom. This term was combined with a range of supplementary key words relevant to nursing education using a PICOS (Population, Intervention, Comparison, Outcomes and Study) framework [19]. The derived terms were: *Population* - Undergraduate Nursing Students; *Intervention* - Flipped Classroom; *Comparison* - Traditional Classroom; *Outcomes* - Educational achievements and pedagogical designs; and *Study* - any original research studies. A search algorithm was created by using keywords with Boolean operators to conduct a literature search in the databases. A sample search strategy in MEDLINE is illustrated in Table 1.

**Table 1:** The MEDLINE search strategy and term used

Search	Algorithms	Article (n)
1	(flip* adj2 (class* or learning or teaching or pedagog*))	483
2	(invert* adj2 (class* or learning or teaching or pedagog*))	205
3	(nursing edu* or nurs* edu* or nurs* or teach* nurs* or health profession* education* or health person* or health person* education* or health occupation* or health occupation*education*)	517,704
4	(undergrad* or baccalaureate or bachelor* or student*)	321,077
5	(Search-1) or (Search-2)	674
6	(Search-3) or (Search-4)	594,770
7	(Search-5) and (Search-6)	413
8	(Search-7) limited to (year = “2012 -Current” and English)	374

### Study selection

Titles of the manuscripts which were identified in the database search were transferred to a bibliography management programme (Endnote X9, Thomson Reuters, New York) to create a search library and remove duplicates. The resulting studies were independently and systematically reviewed by an author (PY) in accordance with the inclusion criteria (Table 2), first by title and then by abstract. Then, full texts of the selected studies were again reviewed by the author (PY) and he made a log of all reviewed studies with reasons for inclusion or exclusion. The log was cross-checked by the other three authors (YC, ML & MAH). Following this, all four authors met at various times to discuss and review all chosen articles. Any disagreements were resolved through discussions within the whole research group until a consensus was reached. Moreover, citations from the selected studies were scrutinised to confirm that all relevant studies were identified.

**Table 2:** Inclusion and exclusion criteria for selecting articles

Inclusion Criteria	Exclusion Criteria
•Description of the Flipped classroom (pedagogy/learning/teaching) in nursing education. •Study using any form of pedagogical model/framework. •Study focusing on measuring the effectiveness of flipped classroom pedagogy. •Study conducted in undergraduate education. •The publication period from 2012 to 2019 (The flipped classroom was introduced into Health Profession Education in 2012 [6]). •Type of publication: Original research, systematic review, or meta-analysis.	•Full text of the article is not published in English. •Study conducted in the context of post-graduate and vocational training. •Study results duplicated in a separate earlier publication. •Type of publication: book, chapters, thesis, commentaries, conference abstracts, protocols, study outlines and government publication.

### Data synthesis

The data synthesis was performed using an electronic data extraction table (in Microsoft Excel). The following details were extracted from each reviewed study: name of authors, country, publication year, participants, research design, research procedure, research instrument, analysis of data, key findings and conclusion. The initial data extraction was completed by PY. The extracted data were independently reviewed for accuracy by the other three authors (YC, ML, & MAH), This group confirmed the inter-rater reliability and resolved any outstanding issues, such as data entry errors. Furthermore, if the details from a selected study was inadequate or ambiguous, additional information was obtained from the corresponding author/s of the relevant study. Lastly, an inductive thematic method was used to analyse the extracted (qualitative) data [20]. This process incorporated a series of inductive stages. First, the extracted data were line-by-line coded by the first author (PY). Then, the codes were crossed checked (by all authors) and clustered under descriptive themes. Finally, the descriptive themes were further condensed into analytical themes to provide an in-depth description regarding the aims of the review. With the exception of the first step, all other steps were conducted in a meeting with the presence of all four authors for establishing inter-rater reliability.

### Quality assessment of the selected studies

An evidence hierarchy classification model (Table 3) was used to assess the quality of the studies [21–23]. Each publication included in the data synthesis was then allocated to an evidence hierarchy classification (I to IV). Subsequently, the publication was assigned to the operational ranks as devised by Jensen et al. (2004) [22]. To maintain the integrity of the quality assessment process, evidence appraisals were independently rated by two authors (PY & MAH). The ratings were presented and discussed amongst all four authors in a meeting to finalize the allocation of category of evidence.

**Table 3:** Categories of evidence and its definitions

Categories	Definitions	Operational ranks
Ia	Evidence from meta-analysis of randomized controlled trials	Rank A
Ib	Evidence from at least one randomized controlled trial	
IIa	Evidence from at least one controlled study without randomization	
IIb	Evidence from at least one other type of Quasi-experimental study	
III	Evidence from non-experimental descriptive studies, such as comparative studies, correlation studies, case-control studies and qualitative studies.	
IV	Evidence from expert committee reports or opinions and / or clinical experience of respected authorities	Rank B-D

## Results

### Study selection

**Fig. 1:**
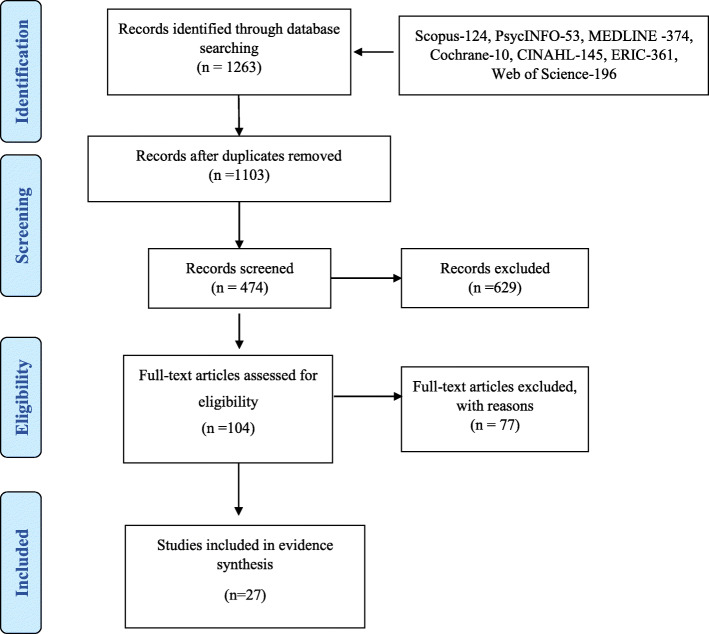
Search methodology PRISMA flow diagram

The initial search yielded a total of 1263 hits from the seven databases (PsycINFO = 53, CINAHL = 145, ERIC = 361, MEDLINE = 374, Web of Sci. = 196, Cochrane =10, Scopus = 124). One hundred and sixty duplicates were identified, and 1103 studies were considered for title and abstract screening. In this title and abstract screening, 629 studies were excluded as they were deemed out of scope. The subsequent quota of studies (*n* = 474) was included for assessing the full texts. A list of 104 studies was identified as potentially relevant to the systematic literature review by three authors. Further, this was reduced to a final list of 27 refereed sources after appraisal of the full texts (Fig. 1). The key study features of the 27 articles in the evidence synthesis are presented in Table 4.

**Table 4:** Studies included in the review

No	Author(s) | Country	Study Design	Academic Year of Sample	Subject Area	Aim(s) of the study	Category of evidence
1	Dehghanzadeh & Jafaraghaee, 2018 [3] | Iran	Quasi-Experimental (Non-equivalent control group Faragher)	2nd Year (*N* = 85)	Musculoskeletal Medical Surgical Nursing	Identify the effect of flipped classroom on nursing students’ critical thinking disposition.	IIa
2	Oh et al., 2019 [24] | South Korea	Quasi Experimental (Pretest-posttest, one group)	2nd Year (*N* = 64)	Nursing informatics	To develop flipped classroom by using film clips and to evaluate the effectiveness.	IIb
3	H. S. Kim, Kim, Cho, & Jang, 2017 [25] | South Korea	Randomized clinical trial	3rd Year (*N* = 62)	Clinical nursing practicum	To develop flipped learning models for clinical practicums and compare their effectiveness regarding learner motivation, satisfaction, and confidence in performing core nursing skills.	Ib
4	Ya-Qian et al., 2018 [26] | China	Meta-analysis	–	–	To examine the effectiveness of the flipped classroom on the development of self-directed learning.	Ib
5	Im & Jang, 2019 [27] | South Korea	Retrospective survey	3rd Year (*N* = 70)	Mental health nursing practicum	To verify the effectiveness of the flipped learning	III
6	Y. M. Kim, Yoon, Hong, & Min, 2019 [28] | South Korea	Quasi-Experimental (Non-equivalent control group pretest-posttest)	2nd, 3th, 4th Year (*N* = 75)	Patient safety course	To examine the effects of flipped classroom on patient safety competency.	IIa
7	Lee & Park, 2018 [15] | Korea	Stratified Group-Randomized Trial	3rd Year (*N* = 102)	Surgical nursing Practicum.	To examine the effect of flipped learning.	Ib
8	Maxwell & Wright, 2016 | [29] USA	Quasi-Experimental (Pretest/posttest control group design)	*N* = 64	Quality improvement and safety	To evaluating the effectiveness of flipped classroom with regard to quality and safety education	IIa
9	Oh, Kim, Kim, & Vasuki, 2017 [1] | South Korea	Quasi-Experimental (Non-equivalent control group pretest-posttest)	*N* = 64	Nursing Informatics	To evaluate the effects of the flipped learning on nursing informatics	IIa
10	Hoover et al., 2018 [12] | USA	Quasi-experimental study	3rd Year (*N* = 42)	–	To examine the readiness for active learning and perceived level of student confidence and preparedness with the flipped classroom method using two different types of pre-class preparation materials	IIb
11	Hew & Lo, 2018 [6] | Hong Kong	Meta-analysis	–	–	To summarize the overall effects of teaching with the flipped classroom approach	Ib
12	Holman & Hanson, 2016 [30] | USA	Descriptive design	*N* = 236	Pharmacology & psychiatric nursing	To analyze the effect of the FM On student learning and to evaluate student perceptions.	III
13	H. Kim & Jang, 2017 [31] | South Korea	Randomized controlled trial	3rd Year (*N* = 202)	–	To verify the effects of flipped learning on the academic achievement, teamwork skills, and satisfaction.	Ib
14	Geist, Larimore, Rawiszer, & Sager, 2015 [32] | USA	Quasi-Experimental (Non-equivalent control group pretest-posttest)	*N* = 86	Pharmacology	To determine difference in content knowledge acquisition between traditional and flipped classroom methods.	IIa
15	Dabney & Mitchell, 2017 [33] | USA	Descriptive study	*N* = 42	Gerontological Nursing	To measure students’ perceptions and satisfaction with the Flipped Classroom.	III
16	El-Banna, Whitlow, & McNelis, 2017 [34] | USA	Crossover repeated measures	*N* = 76	Pharmacology	To examine differences on exam scores and satisfaction of teaching between flipped and traditional classroom approach.	IIb
17	Missildine, Fountain, Summers, & Gosselin, 2013 [5] | USA	Quasi-experimental	*N* = 589	Adult Health nursing	To determine the effects of a flipped classroom and innovative learning activities on academic success and the satisfaction of nursing students.	IIb
18	Simpson & Richards, 2015 [35] | USA	Descriptive and exploratory	3rd Year (*N* = 64)	Population Health	To evaluate the flipped classroom design	III
19	Greenwood & Mosca, 2017 [36] | USA	Quasi experimental design	*N* = 215	Medical-surgical nursing	To determine the relationship between a flipped classroom and test scores	IIa
20	Saunders, Green, & Cross, 2017 [37] | Australia	An exploratory mixed methods design	1st Year	–	To evaluate an integrated flipped and simulated teaching intervention.	III
21	Green & Schlairet, 2017 [38] | USA	Phenomenological approach	*N* = 14	Fundamental Concepts of Nursing	To understand how students perceived their experiences in the flipped classroom and how students’ learning dispositions were affected by the flipped classroom experience	III
22	Bingen, Steindal, Krumsvik, & Tveit, 2019 [39] | Norway	Design-based research	*N* = 192	Physiology	To explore how nursing students experience learning physiology within a flipped classroom.	III
23	Cho & Kim, 2019 [40] | South Korea	Quasi-Experimental (Non-equivalent control group pretest-posttest)	*N* = 80	Clinical adult nursing practicum	To compare the outcomes and influential factors using flipped learning methods	IIa
24	Harrington, Bosch, Schoofs, Beel-Bates, & Anderson, 2015 [41] | USA	Experimental design, randomizing a convenience sample	*N* = 82	Care of the adult	To compare learning outcomes between traditional class and flipped classroom.	Ib
25	Park & Park, 2018 [42] | South Korea	Descriptive and quasi-experimental study	*N* = 81	Adult health nursing	To reveal the effectiveness of flipped learning pedagogy	IIb
26	Hanson, 2016 [43] | Australia	Descriptive research	2nd Year (*N* = 51)	Pharmacology	To examine nursing students’ perceptions of the effectiveness of a flipped classroom	III
27	Xu et al., 2019 [44] | China	Systematic review and meta-analysis	–	–	To examine the effect of a flipped classroom versus a traditional classroom on their skill competence.	I

### Study characteristics

#### Study participants

Participants of the study were defined as undergraduate students who enrolled in the nursing programme. Eleven studies reported the details of students’ academic year of study. Accordingly, the academic year of participating nursing students range from 1st to 4th year. Nevertheless, most of the studies (*n* = 6) were conducted among third-year nursing students (Table 4).

Demographic information of the participants, including age and gender, was included in 12 studies. Two further studies only included the age of participants, while an additional study reported only gender. However, 12 studies did not report demographic variables. The majority of the reported participants were females, and the mean age range was from 19 to 31.5 years. Sample size was reported in the 23 studies which, ranged from 14 to 589.

#### Study setting

The selected studies were primarily conducted in the context of tertiary level nursing education. Most of the studies were conducted in the USA (*n* = 11, 40.7%), followed by South Korea (*n* = 9, 30.3%). Two articles each were found to be published from Mainland China (*n* = 2) and Australia (*n* = 2). One article was published from Norway, Iran and Hong Kong. Moreover, the flipped classroom experiences were reported in reference to a vast range of nursing subjects or courses (Table 4).

#### Methodical quality of studies

According to the evidence hierarchy classification, the majority of the articles (*n* = 19) were IIb (*n* = 5) or above (*n* = 14). The review also included one Ia category evidence [44]. Interestingly, according to operational ranks, all the articles which were included for the review were clustered into rank A (Table 4).

### Evidence synthesis on the flipped classroom in nursing education

#### Qualitative thematic synthesis of findings

**Fig. 2:**
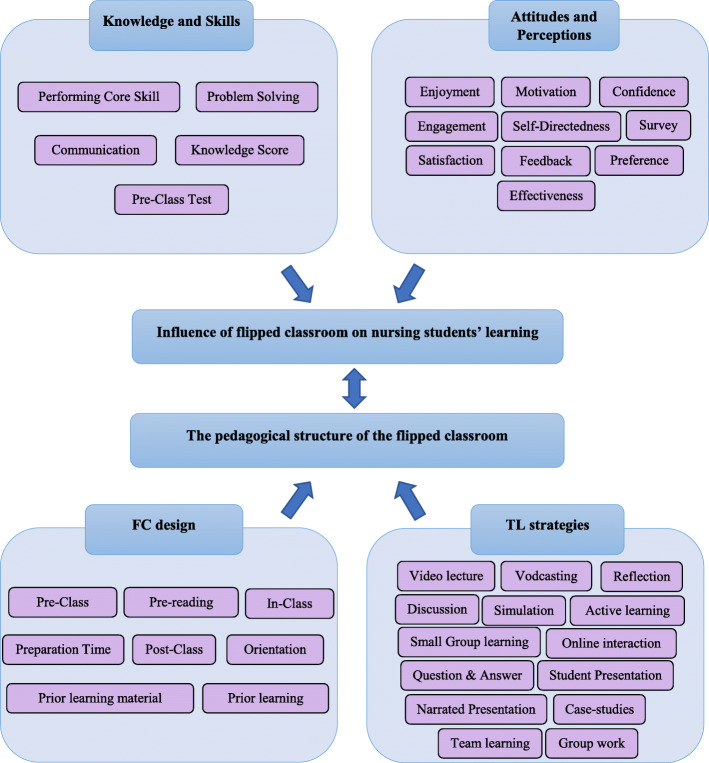
Thematic synthesis

The thematic synthesis revealed 37 codes. The identified codes were clustered into four descriptive themes; namely, knowledge and skills; attitudes and perceptions; flipped classroom (FC) design; and teaching and learning (TL) strategies. The descriptive themes were further specified to two analytical themes for providing profound insights and excelling the context of the present review. The analytical themes revealed were i) the pedagogical structure of the flipped classroom and ii) influence of flipped classroom on nursing students’ learning (Fig. 2).

#### Analytical theme 1: pedagogical structure of the flipped classroom

The systematic review revealed that the flipped classroom is a new educational paradigm in undergraduate nursing education [6, 12]. A common operational flipped classroom model (Fig. 4) was reported in the selected literature which consisted of three components, namely pre-classroom activities, in-classroom activities and post-classroom activities [1, 15, 25, 27, 28, 31, 33, 35, 38–40, 42, 43]. Pre-class activities require the provision of learning material by educators to students [25]. The learning material is mainly transferred through an online learning management system to students [1, 24, 25], at least one week before the face-to-face classroom session [3, 27, 28]. The pre-learning materials can be delivered in various forms, such as video lecture, narrated PowerPoint, animation (illness scenario) and video demonstrations of the nursing procedure [1, 25, 36, 38] with further readings [33, 35, 38, 42]. The duration of the video learning material ranged from 10 to 20 min [15, 27, 31]. Different software were used to create pre-learning materials, such as “Articulate Storyline” [3], “Explain Everything” [27], “Camtasia Studio” [12]. At the end of pre-class activities, an assessment was conducted mainly as quizzes [28, 31, 35, 39]. Furthermore, students were able to interact with teachers and peers through online dashboards [15, 24, 25, 28, 39].

The in-classroom learning environment was designed as an interactive space for applying, analysing and evaluating the pre-learning material [1]. For in-classroom activities, students were divided into small groups [3, 31, 36, 38–40] and the reported group size ranged from two to six [3, 25, 38, 42]. Some studies used quizzes as a diagnostic test at the beginning of the in-class activities [3, 12, 43], followed by several student-centred learning activities [3, 5, 27, 28, 31, 35, 36, 38]. Other studies reported that teachers conducted a micro-lecture for summarizing and clarifying complex phenomena [12, 27, 40, 42, 43].

Post-class activities continued with a follow-up discussion of the newly learnt concepts or issues which had not been solved in the previous in-class session [1, 25]. The follow-up discussions were mainly conducted online [1]. Post-class tests can be conducted to assess students' learning [3, 42]. Finally, at the end of the flipped classroom experience, students completed a survey to evaluate the effectiveness of the flipped classroom [12, 31].

**Fig. 3:**
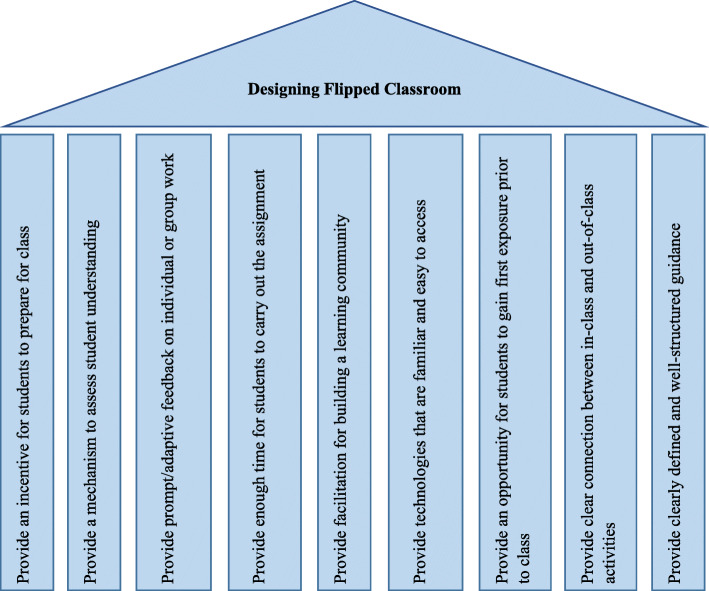
Nine design principles for developing flipped classroom [15]

In terms of developing a flipped classroom, two studies were identified that investigated instructional system designs. Lee and Park (2018) outlined nine design principles for developing a flipped classroom that could be used in a surgical nursing practicum [15]. These are illustrated in the Fig. 3.

**Fig. 4:**
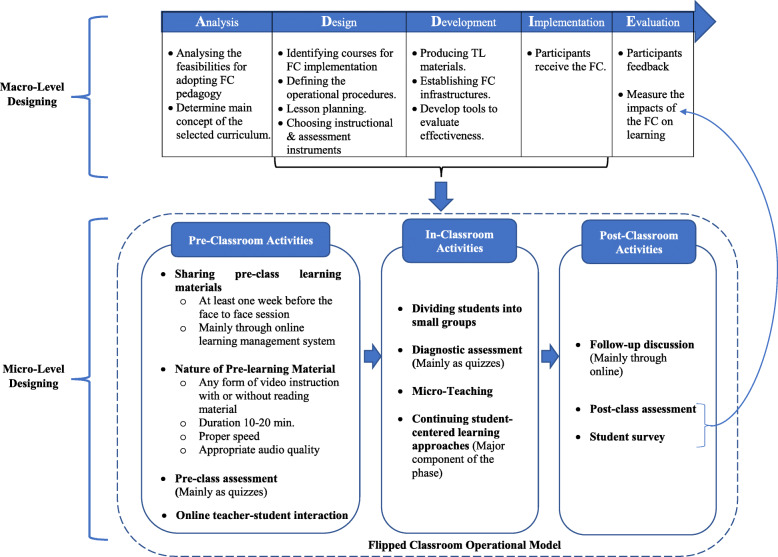
“ADDIE” model and Flipped Classroom Operational Model

Oh et al. (2019) used a framework termed the ADDIE model for developing a flipped classroom to teach a nursing informatics course [24]. This model has been used in terms of its five straightforward steps: Analyze, Design, Develop, Implement, and Evaluate (Fig. 4). First, the Analyze step involves the assessment of feasibilities for adopting flipped classroom in terms of current practice, equipped environment, stakeholder’s readiness and nature of the curriculum. The Design phase deals with framing the instructional strategies, such as identifying courses for implementation, defining the operational procedures, lesson planning, choosing assessment instruments, designing the user interface and choosing the audio-visual designs. The Development phase starts with the production of teaching-learning material for the flipped classroom. In addition, an instrument is developed for measuring the effectiveness of the flipped classroom on students’ learning. The Implementation phase requires participants to receive the flipped classroom. The last step of the ADDIE method is Evaluation. The main aims of this phase are to gather feedback from participants and assess the educational improvement of the learners to quantify the effectiveness of the intervention and identify the way forwards for future improvement [24].

#### Influence of flipped classroom on nursing students’ learning

Several studies addressed the effect of flipped classroom learning on the students’ knowledge and skill. Oh et al. (2019) revealed that integrating flipped classroom led to a significant improvement in nursing students’ test scores [24]. The same impact was observed in another seven studies [5, 28, 29, 31, 32, 36, 42]. In contrast, four studies concluded that flipped classroom learning does not influence students’ assessment performance [30, 34, 35, 41].

Six studies reported the influence of flipped classroom on nursing students’ skill development. Kim and Jang (2017) revealed that students’ clinical performance evaluation score increased after 10 weeks of a flipped classroom intervention [31]. Similarly, nursing students’ core competencies in clinical practicum increased after the flipped classroom experience in a clinical setting [27]. The above two findings were endorsed by a meta-analysis of 22 randomised controlled trials, reporting that flipped classroom improved nursing students’ skill competence [44]. The effect was further confirmed by two studies indicating that the flipped classroom approach increased nursing students’ confidence in performing core skills [25, 40]. Kim et al. (2019) reported that nursing students’ patient safety competency was significantly increased after the flipped classroom experience [28]. In terms of problem-solving skill, Lee and Park (2018) concluded that nursing students who received the flipped classroom showed significant improvements in problem-solving skills [15]. A study investigated communication skills, reported that the outcome of therapeutic communication was significantly increased among the nursing students, after attending the flipped classroom on the mental health nursing practicum [27].

There were several noticeable attitudinal changes among nursing students due to the flipped classroom, namely satisfaction, motivation, engagement, confidence, self-directedness, enjoyment, and critical thinking. In terms of satisfaction, four studies reported that flipped classroom learning increased nursing students’ satisfaction [1, 25, 31, 37]. In contrast, one study noted that nursing students expressed more satisfaction with the traditional lecture-based learning model than flipped classroom learning [30]. In addition, a study reported that nursing students’ satisfaction plummeted at the initial period with the introduction of the flipped classroom [36]. Moreover, nursing students with kinesthetic learning styles were satisfied with the flipped classroom while learners classified as having a preference for visual and auditory stimuli preferred traditional teaching methods [38].

In relation to self-directed learning, four studies reported that flipped classroom pedagogy enhanced self-directed learning among nursing students [1, 26, 30, 40]. Self-goal setting ability was significantly increased among nursing students who attended a flipped classroom [15]. Regarding nursing students’ motivation, two studies indicated that the flipped classroom enhanced learning motivation [1, 26]. In reference to nursing students' learning engagement, the flipped classroom was recognised as an active learning method which enhanced learning engagement [35]. In addition, flipped classroom approaches improved nursing students’ cooperative spirit and teamwork, which increased their interest to engage in learning [44].

Four studies found the flipped classroom an enjoyable way of learning in nursing education [1, 33, 35, 44]. It is interesting to note that nursing students enjoyed viewing the video lecture more than the live lecture [33]. In terms of critical thinking, flipped classrooms increased nursing students’ critical thinking [3, 42]. Moreover, the flipped classroom enabled nursing students’ ability to think deeply and analyse the problem [43, 44].

## Discussion

This systematic review explored and evaluated the flipped classroom in the context of undergraduate nursing education. In particular, the systematic review addressed two main aspects – one focusing on the design and development of flipped classroom pedagogy in undergraduate nursing education and the other evaluating the impact of the flipped classroom on undergraduate nursing students’ learning.

The systematic review identified 27 studies that investigated the flipped classroom experience among undergraduate nursing students. According to the evidence hierarchy classification model [21, 22], most of the selected studies reached the evidence category of IIb or above and all of them achieved operational rank “A”, indicating a catchment of high quality papers. A variety of methodologies, including educational measures were used to determine the impact of the flipped classroom on undergraduate nursing students’ learning. As the studies varied significantly, it is not easy to perform a direct comparison between studies due to the degree of heterogeneity. Nevertheless, the results of the selected studies revealed that a common operational pedagogical structure (Fig. 4) was generally utilised regardless of instructional system designing principles (except for two studies) and the flipped classroom resulted in positive learning outcomes among undergraduate nursing students.

Taking the flipped classroom design into consideration, studies investigated the flipped classroom design in reference to both micro and macro levels [24, 45]. The micro level concerns developing flipped classroom pedagogy for a session or topics [45, 46]. In contrast, the macro level involves instructional system design at the curriculum or course level [45, 47]. Most of the selected studies included in this review examined the flipped classroom at the micro level. Interestingly, the three-step flipped classroom operational model (Fig. 4) describes the flipped classroom design at the micro-level. It was noted that the common operational model was utilised in different forms. For example, Oh et al. (2019) used the basic operational model in eight steps which is called the “C-REVERSE” design with the use of film clips [24] and “flipped-mastery classroom model” was used in the South Korean clinical nursing practicum curriculum [25]. However, the existing findings emphasize that the benefits of the flipped classroom did not eventuate based on sticking purely to the common operational model, but rather caused by the logical connections between the different steps [45, 48, 49].

Some studies included the pre-classroom activities and post-classroom activities under a common cluster of online-learning phase and in-classroom activities labelled as face-to-face learning phase [1, 39, 43, 45, 50]. Three important concerns were reported for developing the online learning phase [45, 46, 51] through evaluating the: (i) physical feature of the video or online lectures which includes duration, pacing and quality of audio; (ii) content feature of the video or online lecture such as appropriate provision of the online portion, clarity and interactivity; and (iii) logistic feature of the video or online lecture namely formative assessment, timetabling and follow-up activities. Designing the face-to-face learning phase is crucial because it is the core part of the flipped classroom [45]. The current review suggests four cardinal activities of the in-class activities, namely dividing students into small groups, conducting a diagnostic assessment, micro-teaching, and continuing integrative student-centred instructions. Furthermore, it has been suggested that the face-to-face learning phase should include: (i) introductory tasks such as mini-lecture and authenticating quizzes; (ii) interactive learning activities which are aligned with the intended learning outcomes; and (iii) well established ground rules and learning culture [45, 46, 52, 53].

In terms of macro-level design, the review identified that the ADDIE model created a framework for designing the flipped classroom for undergraduate nursing students [24]. The ADDIE model has been recognized as effective, systematic and efficient in designing the flipped classroom in nursing education [54, 55]. Moreover, the ADDIE model has achieved acceptance in diverse fields [56, 57]. The ADDIE model proposes five straightforward steps when developing the pedagogical strategies used ensure curriculum planners and implementers reach the ‘appropriate destination’ [56]. In addition, the review traced the design principles of the flipped classroom. Lee and Park (2018) adopted nine design principles (Fig. 3) for developing flipped classrooms in reference to the surgical nursing practicum [15]. Kim et al., (2014) proposed the flipped classroom design principles for enforcing student-centred learning through four key variables, namely cognitive presence, social presence, teaching presence, and learner presence [58].

In reviewing the impact of the flipped classroom on undergraduate nursing students’ learning, positive outcomes were reported in many studies included in this review. More specifically, nursing students’ knowledge, skills and attitudes were improved by the flipped classroom learning, in terms of assessment performance, performing core skills, problem-solving, communication, critical thinking, self-directedness, motivation, engagement, confidence, satisfaction, and joyful learning. Besides, the notions of positive outcomes were reported among students from other discipline such as dentistry, medicine, pharmacy [59–63]. It was reported from the literature that two main explanations contributed to the positive learning outcomes. Firstly, unimpeded access to the pre-classroom learning materials enabled nursing students to learn in their preferred place, pace and time. Specifically, the pre-recorded video lecture was used as the main pre-classroom learning material. The nursing students who watched the video lectures developed a better understanding of learning concepts [6]. Secondly, in-classroom activities were designed as an interactive and student-centred environment which provided greater opportunity to apply the learned concepts into practice [1]. On the other hand, some studies still favoured the traditional lecture-based learning [30, 41]. This may be due to the preference for behaviouristic learning in higher education. Overall, the findings so far seem to suggest that we still have mixed results on whether flipped classroom increases test scores; however, there seems to be strong evidence to suggest that flipped classroom can increase student motivation, satisfactory, and critical thinking.

These findings present two important implications for developing and implementing the flipped classroom in undergraduate nursing education. Firstly, contextual compatibility is more important for the success and sustainability of a pedagogical model. Thus, it is essential to follow an instructional system design at the macro-level to develop flipped pedagogy rather than using its’ operational structures alone at the micro-level. The review identified the ADDIE model and the three-step operational model (Fig. 4) for fostering flipped classroom at the macro and micro level, respectively. Secondly, it was noted that the flipped classroom resulted in positive learning outcomes among nursing students. This outcome may be optimised by balancing the workload of pre-, in-, and post-class activities at the micro level, rather than providing more emphasis on one phase. Furthermore, a study reported that the flipped classroom was not welcome by stakeholders during the introduction phase [36]. Consequently, the solidity of the flipped classroom intervention relies on the constant and stable plan of implementation.

This review could have limitations derived from the heterogeneity of study designs. Apart from meta-analysis, randomised controlled studies, and quasi-experimental studies, we also included several non-experimental descriptive studies to cover the range of available evidence. This heterogenous sample of studies does not permit further probing of the evidence, such a meta-analysis of the study outcomes; however, our sample represents the commonly-used and ethical research methods in educational research and provides a starting point for generating higher levels of evidence. Moreover, the reported findings are mainly from the United States of America and South Korea, which are likely well-resourced settings. Consequently, there may be cultural and regional bias in these studies like ethnocentrism, available resources and educational system. Thus, future research could be conducted in other settings, including low and middle-income countries, to strengthen the evidence base.

## Conclusions

The evidence cited in this systematic review suggests that incorporating the flipped classroom pedagogy probably yields promising positive educational outcomes in undergraduate nursing education. The majority of the studies utilized a common operational flipped classroom structure as pre-classroom, in-classroom and post-classroom. Furthermore, there are promising instructional system design models available for adapting or developing a flipped classroom. Practical implications of the review are considering contextual compatibility and providing equal importance to all three phases of flipped classroom for augmenting the educational outcomes. In addition, the feasibilities of developing the flipped classroom in a limited-resourced setting are still inconclusive. Therefore, future research should consider developing and implementing flipped classrooms for the limited-resourced undergraduate nursing educational environment by using a compatible instructional system designing model.

## Data Availability

Not applicable.

## Abbreviations

PRISMA: Preferred Reporting Items for Systematic Reviews and Meta-Analysis
FC: Flipped Classroom
TL: Teaching and Learning
ADDIE: Analyse, Design, Develop, Implement, and Evaluate
